# Microfabrication approaches for oral research and clinical dentistry

**DOI:** 10.3389/fdmed.2023.1120394

**Published:** 2023-03-09

**Authors:** Paola Tiozzo-Lyon, Matías Andrade, Camila Leiva-Sabadini, José Morales, Antonia Olivares, Andrea Ravasio, Sebastian Aguayo

**Affiliations:** ^1^ School of Dentistry, Faculty of Medicine, Pontificia Universidad Católica de Chile, Santiago, Chile; ^2^ Institute for Biological and Medical Engineering, Schools of Engineering, Medicine and Biological Sciences, Pontificia Universidad Católica de Chile, Santiago, Chile

**Keywords:** microfabrication, organ-on-a-chip, dental research, microarchitecture, 3D printing, photolithography, oral biomaterials

## Abstract

Currently, a variety of laboratory tools and strategies have been developed to investigate *in vivo* processes using *in vitro* models. Amongst these, microfabrication represents a disruptive technology that is currently enabling next-generation biomedical research through the development of complex laboratory approaches (e.g., microfluidics), engineering of micrometer scale sensors and actuators (micropillars for traction force microscopy), and the creation of environments mimicking cell, tissue, and organ-specific contexts. Although microfabrication has been around for some time, its application in dental and oral research is still incipient. Nevertheless, in recent years multiple lines of research have emerged that use microfabrication-based approaches for the study of oral diseases and conditions with micro- and nano-scale sensitivities. Furthermore, many investigations are aiming to develop clinically relevant microfabrication-based applications for diagnostics, screening, and oral biomaterial manufacturing. Therefore, the objective of this review is to summarize the current application of microfabrication techniques in oral sciences, both in research and clinics, and to discuss possible future applications of these technologies for *in vitro* studies and practical patient care. Initially, this review provides an overview of the most employed microfabrication methods utilized in biomedicine and dentistry. Subsequently, the use of micro- and nano-fabrication approaches in relevant fields of dental research such as endodontic and periodontal regeneration, biomaterials research, dental implantology, oral pathology, and biofilms was discussed. Finally, the current and future uses of microfabrication technology for clinical dentistry and how these approaches may soon be widely available in clinics for the diagnosis, prevention, and treatment of relevant pathologies are presented.

## 1. Introduction

Understanding of physiological functions in health and disease continues to be limited by the complexity of biological systems. In recent years a variety of tools and strategies have been developed to investigate *in vivo* processes using *in vitro* models (1). Within this context, microfabrication represents a disruptive technology that, after revolutionizing informatics, telecommunication, and semiconductor industries, is currently enabling next-generation biomedical investigation through the development of complex laboratory approaches (e.g., microfluidics), engineering of micrometer scale sensors and actuators (micropillars for traction force microscopy), and creation of biomimetic (*in vivo*-like) environments recapitulating cell-, tissue-, and organ-specific contexts (2). The term microfabrication, especially in the field of bioengineering, refers to a set of processes to control the fabrication of substrates and devices where at least one of the feature dimensions is in the micro- or nanometer scale. Generally, two strategies are used to achieve this spatial control (3). Bottom-up approaches leverage from the self-assembly properties of molecules and polymers to build up into structures of controlled geometries (e.g., atomic layer deposition, sol-gel fabrication, vapor deposition, etc.). These technologies are most suited to fabricating nanoscale structures such as surface nanotopographies. On the other hand, top-down approaches make use of various fabrication strategies whereby structures are fabricated by removing parts till the desired geometries are achieved. This typically refers to methods such as photolithography and etching, but it has been used to describe downstream processes such as soft-lithography and high-resolution 3D printing (4). In the biomedical field, microfabricated materials can be used to study e.g., the role of surface topographies on cell adhesion, differentiation, and signaling (5, 6). Furthermore, the use of micro- and nano-fabricated biomaterials is finding its way into clinical practice with great impact on tissue regeneration, nanocarrier fabrication for the delivery of therapeutic agents, extended implant lifespans, bacterial adhesion and growth prevention, and design of novel diagnostic tools for personalized medicine, amongst others (7, 8). In this review, we aimed to deliver an overview of the current understanding of microfabrication approaches in dental studies and clinical practices. In addition, we will provide a perspective guideline for currently unexplored instances where microfabrication could prove to be a game-changer in the field.

## 2. Microfabrication approaches in oral and dental research

**Figure 1:**
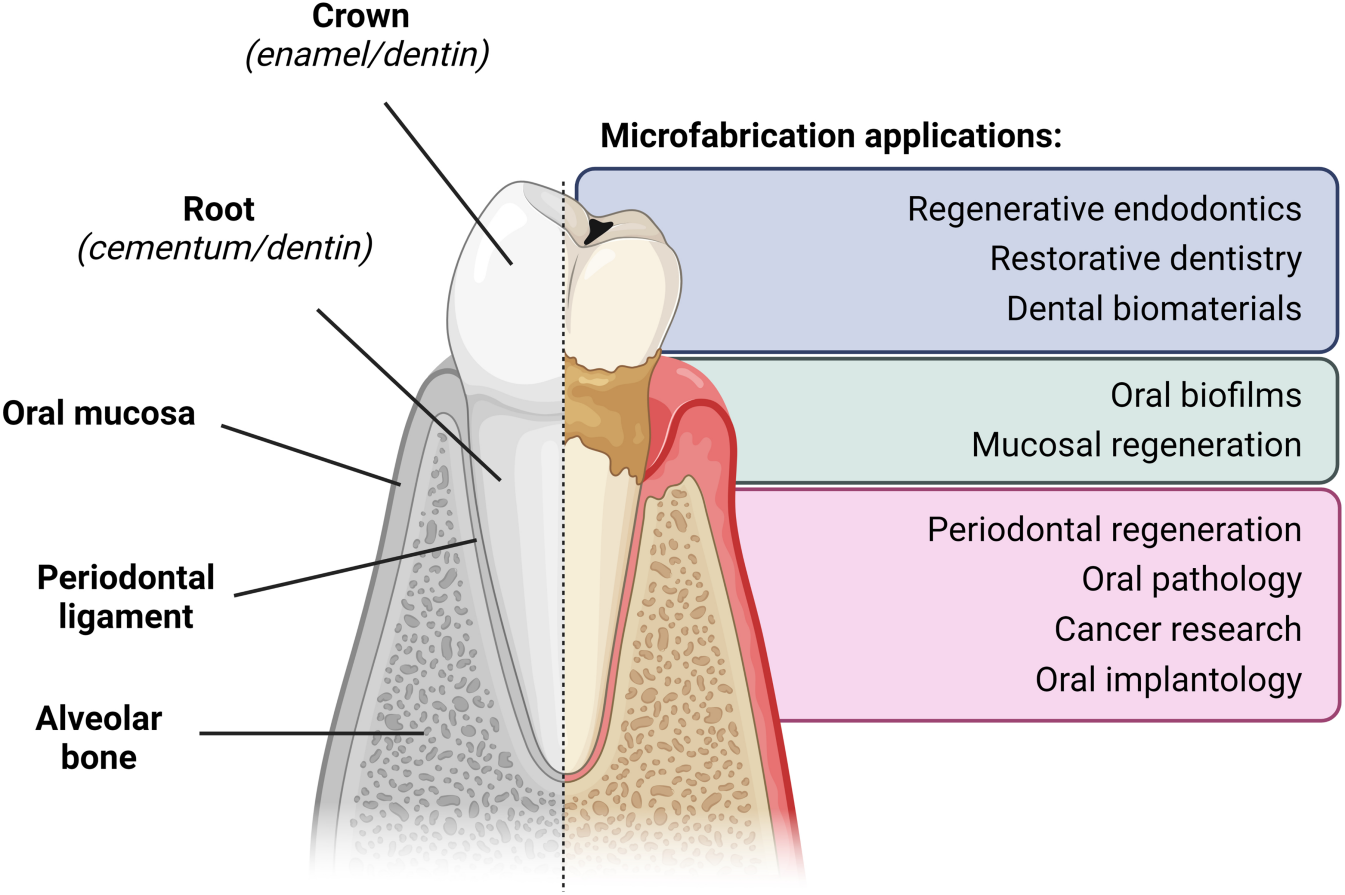
Summary of current research applications of microfabrication in dental research. Spatial architecture of the dental and periodontal tissues, as well as the main current applications of microfabrication-based approaches for the study of oral diseases and conditions.

Although microfabrication has been around for a number of years, its application in dental and oral sciences is quite recent. Currently, multiple lines of research have emerged in oral sciences that use microfabrication-based approaches for the study of oral diseases and conditions, as well as for use in clinically relevant applications such as diagnostics, screening, and biomaterial manufacturing (Figure 1). Indeed, microfabrication approaches are particularly suited to recapitulate the structures and micro-architectures found in the tooth, consisting of only one non-mineralized (dental pulp) and three mineralized (enamel, dentin, and cementum) tissues (9). Furthermore, teeth are surrounded by periodontal tissues that are rich in extracellular and cellular components that have very particular microarchitectures (e.g., such as the cementum-periodontal ligament-bone interface) (10, 11). On the other hand, dental caries and periodontal disease are two of the most prevalent human pathologies worldwide, both of which result in the irreversible destruction of tissues (12, 13). Therefore, there is a continuous need to develop novel tissue-specific approaches for local regeneration as well as to create new, smart bioactive materials and drug carriers. Hence, it is no surprise that microfabrication is being explored as a potential way to fabricate novel biomaterials and scaffolds that replicate the extracellular matrix of native oral and dental tissues with micro- and nano-scale sensitivity (14).

Despite their increasing use in many biomedical fields, the advent of microfabricated biomaterials and devices in dental research is still quite new, and not much is known about their potential use for cutting-edge research in the oral and craniofacial fields. As a result, we have carried out a search of PubMed, Web of Science, Scopus, and Google Scholar for literature on microfabrication associated with dentistry and dental sciences. Thus, this review summarizes and discusses the most relevant applications of microfabrication in oral sciences for both research and clinics.

### 2.1. Commonly used microfabrication strategies

In recent years, a number of microfabrication methodologies have been investigated for application in biomedical research (Table 1). One of these approaches is lithography which allows for the development of structured surfaces at the micrometer and nanometer scales (15). Photolithography involves the transfer of a computer-designed pattern to a surface of interest, which is then used as a guide to modify the material by further processes such as photopolymerization or etching (16). One of its advantages is the ability to generate patterns with defined 2D micrometric geometries (17, 18). Electrospinning is another technique used in biomedical sciences where a fiber pattern is generated by ejecting a polymer solution under a high-voltage electric field into a metallic collector (28). Due to their cost-effective fabrication, high surface area, and tailored porosity, electrospun fibers have been used in oral sciences as tissue engineering scaffolds, wound dressings, and drug delivery systems (29). Furthermore, as electrospun scaffolds can be loaded with molecules, growth factors, nanoparticles, and pharmacological agents, their potential as vehicles for drug delivery can open a wide range of novel applications in dentistry in the future (29).

**Table 1:** Commonly used microfabrication approaches in oral and dental research.

	Illustration	Short description	Advantages	Materials	Applications in dental research	Reference
Photo- and soft-lithography	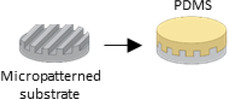	Pattern transfer onto a biomaterial substrate	Lower costs, controlled micrometer morphologies	Silicon wafers, titanium, and organosilicon polymers	Cell sorting and classification, microbial bioimaging, implant surface modification	(15–18)
Microfluidics	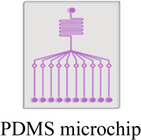	Microfabricated structures for small fluid volumes	High specificity, quick sample processing, cost reduction	Polymers (e.g., PDMS)	Lab-on-a-chip and organ-on-a-chip systems for diagnostics and screening	(19–23)
3D bioprinting: fused deposition modeling	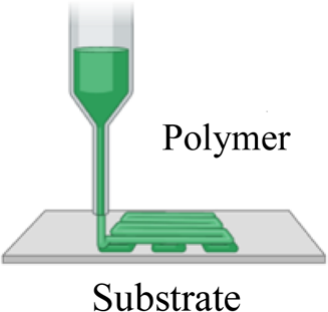	Fabrication by extrusion of thermoplastic material	Fast, high-quality production of biomaterials	Polymers	Oral and maxillofacial surgery, fabrication of fixed and removable prosthetics	(24)
3D bioprinting: laser stereolithography	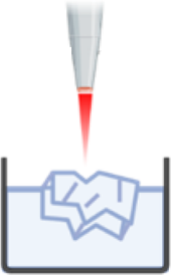	Solid free-form fabrication by additive manufacturing	High microscale precision and rapid manufacturing	Polymers, hydrogels, resins	3D printed diagnostic and therapeutic models, implant fabrication	(25)
Microspheres	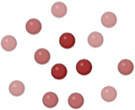	Spherical polymerized networks	Tailored fabrication according to need, easy fabrication	Organic or inorganic polymers	Targeted drug delivery, molecule carriers, cell expansion *in vitro*	(26, 27)
Electrospinning	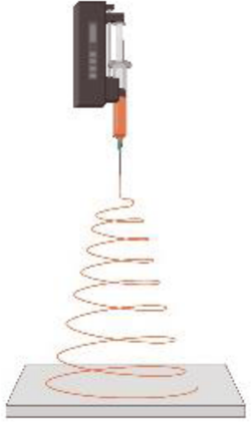	Production of polymer fibers by electric force	Cost-effective scaffold production in the micro- and nano-scale range	Natural, synthetic, and semi-synthetic polymers	Tissue engineering scaffolds, drug delivery materials, novel restorative biomaterial reinforcement	(28–30)

Other microfabrication techniques of interest include three-dimensional (3D) printing *via* fused deposition modeling or laser stereolithography, methods that allow the creation of biomaterials *via* additive manufacturing (24, 25). In these approaches, 3D materials are designed in specialized software and divided into a series of 2D layers with an equal thickness (31), which are then printed layer-by-layer until the full material is completed (32). Furthermore, the fabrication of microspheres for the bespoke delivery of drugs and molecules has also been explored with potential uses in bone and tissue regeneration (26, 27).

Microfluidic chip-based models, fabricated *via* soft lithography and molding, typically consist of polydimethylsiloxane (PDMS) platforms presenting channels and reservoirs that allow fluids to be controlled and manipulated at the microscale (19–21). These chips provide several advantages including precise control of experimental conditions (e.g., flow, the concentration of chemical species, rate of chemical reactions) while allowing inspection of biological samples *via* microscopy and reducing laboratory costs by decreasing the number of reagents needed for each experiment (17). Perhaps the most promising avenue in microchip-based models is the development of *lab-on-a-chip* and *organ-on-a-chip* systems in biomedicine and dentistry. On the one hand, *lab-on-a-chip* approaches can be used as diagnostic tools for small samples of a bodily fluid such as blood, saliva, or urine, in order to identify proteins, hormones, or pathogens, among others (22). On the other hand, *organs-on-a-chip* are intended to replicate the environment and physiology of a particular tissue or organ in a complex three-dimensional *in vitro* model (23). Therefore, these systems are being used to study the physiology of tissues and organs of interest, as well as specific pathologies and potential pharmacological treatment of these conditions. To date, microfabricated chips have been manufactured to replicate the function of organs such as the pulmonary system, cardiovascular system, brain, liver, and kidneys, among others (22).

In addition to the above, there remains an interest in integrating different organs on a chip into one overall system to evaluate the interaction between different organs in the laboratory. This approach, also known as *human-on-a-chip* or *multiorgan-on-a-chip*, would have the potential to study not only the therapeutic effect of a drug in a certain target organ but also evaluate its potential toxicity to other relevant organs (33). However, the fabrication of these models is a great challenge due to the difficulty of integrating different systems on a microfabricated chip in an effective and cost-efficient way (34).

### 2.2. Endodontics and dental pulp regeneration

When a tooth is subjected to acute damage such as a carious lesion, trauma, or dental fracture, it can cause irreversible inflammation or necrosis of the dental pulp. Given this scenario, one of the most utilized therapeutic options is endodontic treatment; however, several complications may arise including disruption of the mechanical properties of the tooth that significantly increases fracture risk (35). On the other hand, a tooth loses its sensory capacity after endodontic treatment; therefore, any secondary caries lesions on the tooth may not generate symptoms and complicate the detection of these pathologies in a timely manner. Faced with these limitations, pulp regeneration strategies were proposed many years ago as a therapeutic alternative in cases of irreversible pulpitis or pulp necrosis. Nevertheless, there are multiple limitations that hinder its widespread implementation in clinics, such as the limited vascularization of the tooth that hinders pulpal regeneration cell viability post-intervention (36). To overcome these difficulties, a range of microfabrication techniques are being explored as possible alternatives for the study of pulpal regeneration as well as for the development of new clinical approaches (Table 2).

**Table 2:** Main microfabrication approaches in oral and dental research.

	Main current and potential uses in research and clinics	Microfabrication techniques	References
Endodontics	- Scaffold and microspheres for dental pulp stem cell growth - Growth factor delivery	- Electrospinning and scaffolds - 3D bioprinting - Microspheres	(37, 38) (39) (40)
Restorative Dentistry	- Cytotoxicity and antimicrobial assays for oral biomaterial testing	- Microfluidics and tooth-on-a-chip - Electrospinning	(41, 42) (30, 43)
Periodontal regeneration	- Promotion of cell adhesion and collagen deposition - Epithelial regeneration - Selective PDL/bone regeneration - Drug delivery	- 3D bioprinting and lithography - Scaffolds - Tooth-on-a-chip - Microspheres	(44, 45) (46) (47) (48)
Dental implants	- Design of microstructured implant surfaces for improved cell adhesion - Microsensors for monitoring real-time periodontal inflammation	- Photolithography	(49–52)
Oral microbiology and biofilms	- Real-time biofilm formation - Antibiotic resistance evaluation - Bacterial adhesion to surfaces	- Lithography and microfluidics	(53–55)
Oral cancer	- Early oral cancer screening - Tumoral cell detection in saliva	- Microfluidics and lab-on-a-chip	(56, 57)

Within the literature, the manufacture of scaffolds made of both natural and synthetic polymers in which dental pulp stem cells can be cultured has been reported (37). The microfabrication techniques used to manufacture these scaffolds include electrospinning, 3D printing, self-assembly, and micro/nanosphere systems (39). Amongst these, Li et al. designed a microsphere-based injectable system that acts as a scaffold for dental stem cell proliferation (40). Here, researchers manufactured a scaffold system made of biodegradable polylactic acid (PLA) microspheres which contained heparin-conjugated gelatin nanospheres containing vascular endothelial growth factor (VEGF). This system acted as both a transport and scaffolding medium for dental pulp stem cells, while at the same time acting as a sustained release system for VEGF over time (40). Furthermore, the fibrillar structure and high porosity of these materials were comparable to the extracellular matrix of collagen. Finally, the authors noted that in this system, the degradation and absorption of microspheres produced a sustained and controlled release of VEGF when injected into the root canal of extracted human teeth and subsequently implanted into an animal model. After 9 weeks, the proliferation and differentiation of dental pulp stem cells toward odontoblasts were observed, accompanied by a large number of blood vessels throughout the pulp (40). Furthermore, Albuquerque et al. have developed an antibiotic-containing PDS fiber scaffold using electrospinning. Metronidazole, minocycline, and ciprofloxacin were incorporated into the scaffold and tested against endodontic pathogens such as *Enterococcus faecalis* and *Actinomyces naeslundii*. The scaffold had the ability to sustainably release the antibiotic mixture causing bacterial death without affecting dental pulp stem cell proliferation and migration (38). However, studies regarding the use of microfabrication in dental pulp regeneration are mostly restricted to *in vivo* or *in vitro* experiments, so the extrapolation of these results into the clinical setting remains quite limited. In the future, researchers hope to use these and other microfabrication approaches—such as vasculature engineering—to generate scaffolds that promote dental pulp revascularization and regeneration after injury or restorative treatment with predictable outcomes (58).

### 2.3. Restorative dentistry

In dentistry, the interaction between biomaterials and oral tissues is a crucial component for biocompatibility and the long-term success of dental restorations. However, many difficulties remain in order to correctly replicate the *in vivo* tissue-biomaterial interface in the *in vitro* setting. To solve this problem, França et al. have been pioneering the implementation of microfluidic-based systems to replicate the physiology of dental pulp and its interaction with different biomaterials (41). For this, they designed a device called “tooth-on-a-chip” by using a combination of PDMS and polymethylmethacrylate (PMMA) by employing 3D printing and microfabrication. By designing two parallel channels separated by a block of human dentin, stem cells from the apical papilla were co-cultured with different dental biomaterials to replicate the pulp-dentin-biomaterial interface and evaluate the pulp response in real time. In an initial publication, they evaluated the cytotoxicity of materials used in restorative dentistry such as hydroxyethyl methacrylate, resin-based adhesive systems, and phosphoric acid by employing their tooth-on-a-chip system (41). Subsequently, in a second study, the authors used the same device to evaluate the effect of different calcium silicate-based cements and their ability to induce cell proliferation in pulp cells (42). Authors observed differences in cell proliferation, viability, morphology, transforming growth factor-β expression, and antimicrobial activity according to the employed biomaterials (42). Overall, these microfluidic systems are highly reproducible *in vitro* platforms for the high-throughput evaluation of biomaterials for restorative dentistry; however, clinical studies are still needed to validate the *in vivo* effectiveness of these approaches. Nevertheless, the further development of tooth-on-a-chip devices shows great promise for the real-time evaluation of biomaterials, molecules, and drug therapies against cells and biofilms of clinical interest (Table 2).

Furthermore, microfabrication techniques are gaining traction as a tool for the development of dental materials such as fiber-reinforced composites, which have shown improved mechanical properties compared to regular-filled composites (43). In Tian et al., the incorporation of nylon 6 fibers into a resin matrix was achieved with electrospinning, resulting in spun nylon 6 nanofibers with an average of 250 nm diameter. The fiber was then milled and incorporated into a resin matrix in various mass fractions (1%, 2%, 4%, and 8%) to observe their effect on the mechanical properties of the resulting biomaterial. Results demonstrated an increase in flexural strength, elastic modulus, and work of fracture for the small mass fraction groups (1% and 2%) (30). Similar to the previously discussed literature, this work is also mostly focused on *in vitro* formulations and testing, and further efforts must be made to validate the use of these microfabricated biomaterials in the clinical setting.

### 2.4. Periodontal regeneration

The use of microfabrication has also potentiated cutting-edge research on periodontal regeneration (Table 2). Periodontal tissues such as the alveolar bone, periodontal ligament (PDL), and oral mucosa are highly complex, and as such, there is a need to develop small-scale biomimetic biomaterials to effectively regenerate areas with extended tissue destruction following periodontal disease (59). To do so, some groups have utilized 3D-printed microfabrication to generate surfaces with micropillar cues to promote the deposition of aligned collagen fibers for PDL regeneration (44). After seeding with PDL cells, authors observed cellular alignment and the deposition of orientated collagen amongst the micropillars in an animal model, showing promise as an approach for the guided reconstruction of soft tissues (44). Furthermore, Suzuki et al. fabricated a collagen membrane to be used as scaffolding for both extraoral and intraoral epithelial regeneration by mimicking the shape and distribution of dermal papillae (46). Firstly, the authors employed lithography to generate microfabricated surface topographies in order to mimic the tissue pattern of the epidermis and dermis. Subsequently, a tilapia collagen mixture was poured into the molds and further crosslinked in order to improve the mechanical properties of the membrane (46). Overall, the authors observed epithelial regeneration directly associated with the membrane structure and suggest that this technique may have the potential to function as a membrane that allows cell regeneration in a biomimetic way.

In other work, Lee et al. have focused on creating microstructured materials with region-specific properties to simulate the cementum-PDL-alveolar bone complex for optimized periodontal regeneration (45). For this, authors engineered specific microarchitectures for each region and selectively loaded the scaffold with either amelogenin, connective tissue growth factor, or bone morphogenic protein to promote cementum, PDL, and alveolar bone regeneration, respectively. These micro-channeled substrates were generated *via* 3D bioprinting and were found to promote distinct cell differentiation, collagen deposition, and mineralization within the construct in both *in vitro* and *in vivo* experiments (45). On the other hand, Vurat et al. designed a microfabricated model to replicate the interface between the PDL and alveolar bone, similar to a tooth-on-a-chip system. The authors used 3D bioprinting to fabricate hydrogel blocks containing human periodontal ligament fibroblasts and osteoblasts. Subsequently, both hydrogels were united and the resulting microtissue was included in a PDMS chip containing a circuit that allowed the diffusion of culture media to monitor cell viability and migration over time (47). The authors used tetracycline as a model drug for the antibiotic treatment of periodontal disease and were able to measure its uptake by the scaffold-encapsulated cells. Overall, the use of these models to study the response of periodontal and alveolar bone cells to drug loading or bioactive molecules is of great interest for future studies on the treatment of periodontal diseases. However, more clinical research is still needed in order to translate these systems into clinics and patient care.

Furthermore, oral bone regeneration is also an area that has gained much interest due to the increased use of dental implants for tooth replacement. Currently, the treatment for bone defects is mainly based on the use of bone grafts that provide an osteoconductive media for regeneration. Therefore, the complementation of bone regeneration with microfabricated osteoinductors such as growth factors is currently being explored. In this context, Ma et al. developed an injectable system of poly (lactic acid) (PLA) microspheres carrying gelatin nanospheres bound to bone morphogenetic protein (BMP-2). This system allowed both improved cell adhesion and a sustained release of BMP-2 from the microspheres over time (48). There is also a growing interest in the use of scaffold-free approaches for regenerative periodontics and endodontics, including the use of extracellular vesicles, spheroids, and tissue strands that aim to promote the development of a biomimetic extracellular matrix for regeneration (60). The microfabrication of tissue-specific stem cell niches that promote local tissue regeneration is also of great interest (8). Nevertheless, most of these investigations remain strictly *in vitro* and challenges remain in order to translate these microfabricated technologies and biomaterials into standardized and reproducible clinical approaches, particularly as the microarchitecture of the periodontal region is highly complex compared to other tissues.

### 2.5. Dental implant research

A crucial element for the success of titanium dental implant treatments in dentistry is the correct integration of the implant into the periodontium (including the alveolar bone) and the surrounding soft tissues. To achieve this, it is known that the surface topography and roughness of the implant are important factors to achieve intimate contact between the alveolar bone and the titanium surface (61). Therefore, it is no surprise that the use of microfabrication techniques has been implemented to attain biologically active titanium surfaces (Table 2) (62).

For example, Doll et al. have published different strategies to design and fabricate surface patterns on titanium frameworks (49). They initially proposed a subtractive method by acid etching using photolithography. However, as the substrates in which lithography techniques are used are usually flat, a flexible photomask was manufactured to apply this technique onto a dental implant surface by using a mechanism allowing rotation of the implants while they are exposed to ultraviolet light (49). By employing this method, they obtained microscale features up to 1.5 µm sizes with the hope of improving fibroblast and epithelial cell adhesion to dental implants, in order to increase soft tissue interactions with dental implant biomaterials in the future.

On the other hand, the same group of researchers published a proposal for an additive method using a photolithography method. However, instead of using chemical etching, they proposed the use of anodic oxidation in which an oxide layer is formed on the titanium surface (50). Furthermore, in a recent investigation, Moreira et al. developed micropatterned silica coatings consisting of either lines or micropillars to increase osteointegration in zirconia dental implants (51). The resulting surfaces displayed increased hydrophilicity compared to controls and therefore showed great promise to promote cellular attachment and long-term osseointegration.

Microfabrication has also been used to generate real-time sensors for monitoring inflammation surrounding dental implants. In this context, Kim et al. developed a temperature-based polymer microsensor to monitor implant survival and predict potential failures (52). By using photolithography, authors fabricated polymer microfilms that were wrapped around dental implant abutments and were able to sense temperature changes in the surrounding environment. The microsensor sensitivity paired with its adequate mechanical and chemical resistance suggests its potential use in clinics to develop personalized diagnoses and long-term follow-up for dental implant treatments.

### 2.6. Oral microbiology and biofilm sciences

The formation of multispecies biofilms in the oral cavity is an important problem in dentistry as they play a key role in the development of diseases such as dental caries, periodontitis, and peri-implantitis (63–65). More specifically, the dysregulation of the oral microbiota (dysbiosis) due to the over-proliferation of certain pathological strains is associated with site-specific ecological changes (66). Until now, the incubation of microorganisms in closed systems under static conditions where nutrients are depleted, and waste products accumulate has been the most utilized approach. Nowadays, however, thanks to microfabrication it is possible to assess biofilm formation on surfaces using microfluidic devices that simulate different hydrodynamic flow conditions (67). Furthermore, microfabricated systems can be used to study the effect of antibiotics and antimicrobial molecules on bacteria, as well as the resulting changes that occur at the bacterial and biofilm levels (Table 2) (68).

Within this context, there are several approaches to characterize bacterial adhesion and biofilm formation using microfabrication-derived approaches. For example, Straub et al. monitored bacterial adhesion in real-time using a microfluidic system coupled with optical microscopy, observing how medium composition can impact biofilm formation (53), and Tang et al. utilized a microfluidic chip to explore the biofilm dynamics of antibiotic-resistant *Escherichia coli* (54). More specifically in dentistry, Alvarez-Escobar et al. have recently employed lithography techniques to study intraoral bacterial adhesion to substrates (55). Authors designed PDMS plates with different surface patterns that were subsequently incorporated into intraoral retainers for 24-hour *in vivo* bacterial adhesion and biofilm formation. Although no significant differences in biofilm formation were observed among substrates, this pilot study proposes a method to study bacterial adhesion directly onto different dental biomaterials or dental tissue specimens (55).

### 2.7. Oral cancer research

Finally, microfabrication and microfluidic techniques are also being developed as novel low-cost screening strategies for oral cancers, particularly with the objective of early detection and treatment (Table 2). Proof of concept and early designs for lab-on-a-chip-based microfluidic systems looked to automatize the detection of certain saliva-based markers for oral squamous cell carcinoma (56). However, more recently, Zoupanou et al. designed a microfabricated chip with the ability to immobilize circulating tumor cells (57). To do this, a PMMA microfabricated chip containing a microfluidics circuit was constructed using laser ablation techniques and further treated to bind epithelial cellular adhesion molecule (EpCAM), an important membrane biomarker expressed by tumor cells of epithelial origin. By using this system, researchers were able to effectively immobilize circulating tumor cells with the aid of anti-EpCAM antibodies and demonstrated the potential of this system as a plug-and-play device for rapid and easy oral cancer screening (57).

## 3. Current uses of microfabrication in clinical dentistry

**Figure 2:**
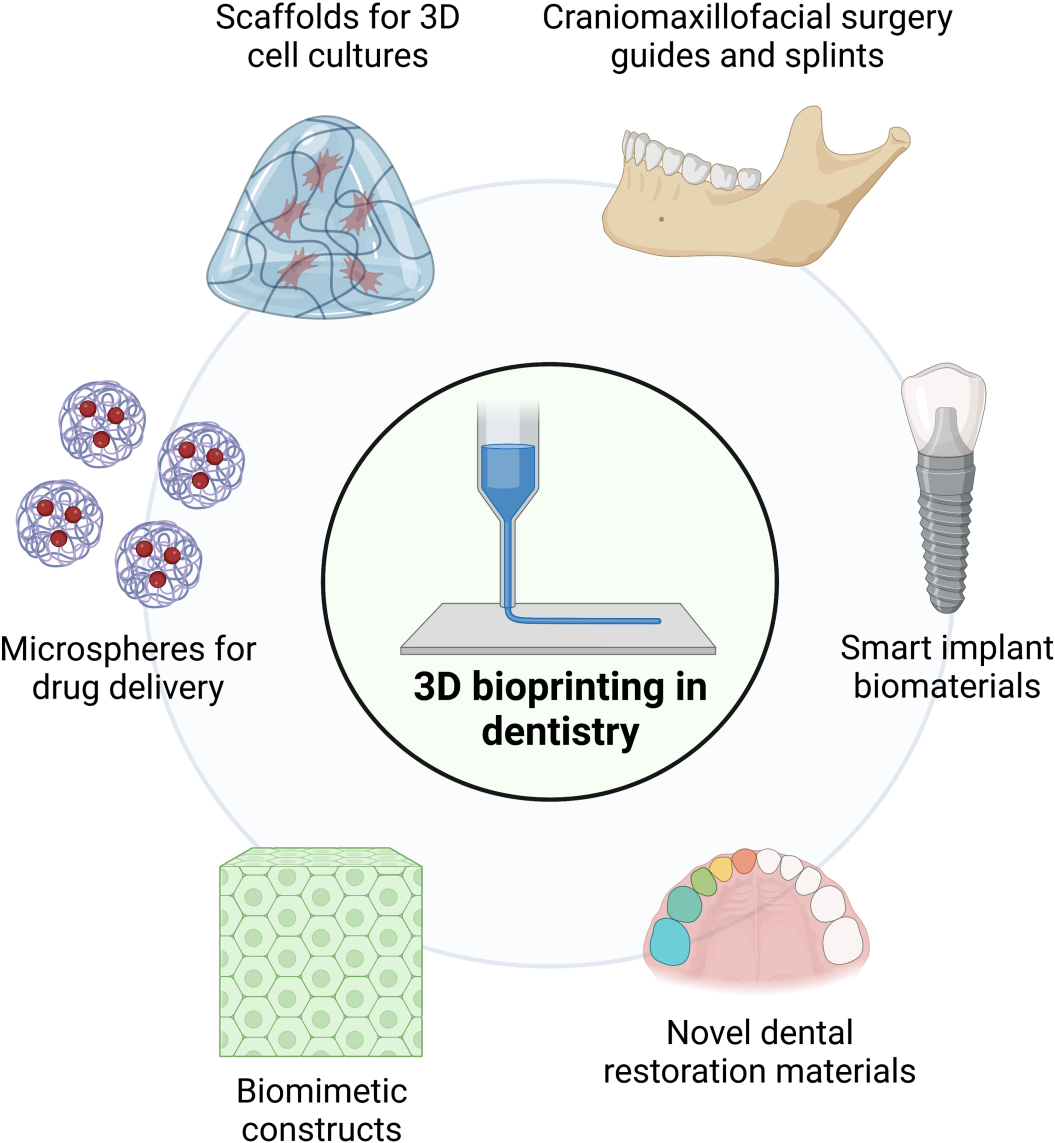
Current and potential clinical uses of microfabrication and 3D bioprinting in dentistry.

Until recently, microfabrication approaches in dentistry have been mostly utilized in the *in vitro* setting for the study of relevant oral diseases and conditions. However, there is a growing interest in the use of microfabrication-based techniques and methods for clinical applications in a variety of dental disciplines (Figure 2). Among these techniques, 3D bioprinting has gained a lot of traction within clinical dentistry (69, 70) and is currently being explored in the fields of prosthodontics, maxillofacial surgery, orthodontics, endodontics, and periodontics (71, 72). Furthermore, the fabrication of 3D printed materials with microscale topographies seeks to promote the biological interaction of biomaterials with human tissues to improve the long-term prognosis of dental treatments in clinics (73).

Currently, the process of combining cells with 3D-printed polymers to create 3D cell cultures for tissue engineering, drug screening, or *in vitro* disease models is increasing its popularity (74). Microfabrication approaches based on spheroid/microtissue systems have given rise to potential applications for the clinical regeneration of damaged tissues as well as the construction of *in-vitro* tissue models to understand cell behavior (75). Many methodologies utilize scaffolds manufactured from natural or synthetic polymers such as collagen (76), or PLA and poly (glycolic acid) (PGA), respectively (77). The scaffolds then act as templates that allow cells to adhere, proliferate, and expand throughout the 3D matrix and eventually generate mature cell-laden grafts with features comparable to native tissue. Studies have shown that the phenotype of scaffold-seeded cells can be regulated by a combination of different biological and physical stimuli (74, 78, 79). Additionally, 3D bioprinting technology offers the unprecedented ability of engineering biomaterials that mimic the shape, structure, and function of native tissues and organs (80). The resulting 3D-printed biomaterials are expected to serve as biomimetic constructs that ensure increased cell viability and support of tissue and organ functionality (81, 82). Recently, a couple of case reports have pioneered the use of 3D bioprinting for the treatment of periodontal defects (83) and alveolar cleft reconstruction (84); nevertheless, larger clinical trials are necessary in order to determine the long-term success of these approaches for reliable tissue regeneration.

3D printing has several advantages compared to traditional approaches, mainly due to the rapid and high-precision production and customization of biomaterials with microscale sensitivity (85). In addition, 3D printing provides personalized service at a lower cost for patients and thus simplifies the complex workflow related to the production of some dental appliances (69). From a clinical perspective, the use of 3D-printed restorations has shown notable advantages, such as reduced internal and marginal gaps compared to milled restorations (70) and rapid manufacturing of complex geometries (86). From the biomaterial perspective, a variety of materials can be used to create complex geometric shapes and precisely meet dental needs, especially when combined with 3D scanning of the patient's tissues. For example, advances in intraoral and extraoral 3D scanning technologies, cone beam computed tomography (CBCT), and other CAD/CAM technologies have fueled the use of 3D printing in the field of oral and maxillofacial surgery (87–89). On the other hand, temporary crowns obtained by 3D printing have shown greater mechanical resistance since their construction eliminates operator-induced errors (90). Furthermore, dental crowns and bridges can be manufactured using 3D printing technology (91), and in some cases, a variety of materials can be printed simultaneously with favorable detail reproducibility (92). In the case of removable prostheses, resin bases can be generated without the need for impressions or cast models (86). However, these technologies are not yet widely available in clinics and there remains a lack of long-term clinical studies assessing the durability and biological effect of 3D-printed dental biomaterials.

It is known that proper tissue adaptation is critical for removable denture stability and retention (93) and in this context, Tasaka et al. found that 3D printed dentures had a higher precision than those produced by conventional thermal polymerization (94). Overall, resin-based 3D printing involves the use of photosensitive materials that are cured and molded under light irradiation (95). More specifically, these resins are deposited on a model-building platform and cured with an ultraviolet (UV) laser to generate a morphology of interest according to a computer-generated design (96). Therefore, these approaches can generate a wide variety of material densities, hardness, flexibilities, and porosities. Furthermore, surface printing resolutions in the tens-of-microns range together with the fabrication of complex geometries are important advantages of these approaches compared to conventional prosthesis manufacturing (97, 98).

Additionally, there is much research centered on using 3D bioprinting to fabricate dental implant biomaterials with micrometer features to promote tissue integration. Recent work by Yin et al. employed 3D-printed implants with micropore channel architecture to improve alveolar bone height preservation and promote cell differentiation and actin remodeling (99). Further investigations have functionalized polymer biomaterials with chlorhexidine-containing silica nanoparticles embedded in PDMS to confer antibacterial properties (100). Also, 3D bioprinting is currently being explored to generate microporous carbon fiber and hydroxyapatite constructs to enhance the toughness and strength of scaffolds for bone regeneration (101). However, most of these materials remain still in the developmental phase, with investigations limited to *in vitro* mechanical characterization and pre-clinical testing. However, it is expected that soon there will be a strong increase in available biomaterials and equipment to use 3D bioprinting in the clinics, particularly associated with microscale and nanoscale topographies and geometries to promote biological activity and long-term integration to the host tissues.

## 4. Future trends and opportunities for microfabrication in dentistry

**Figure 3:**
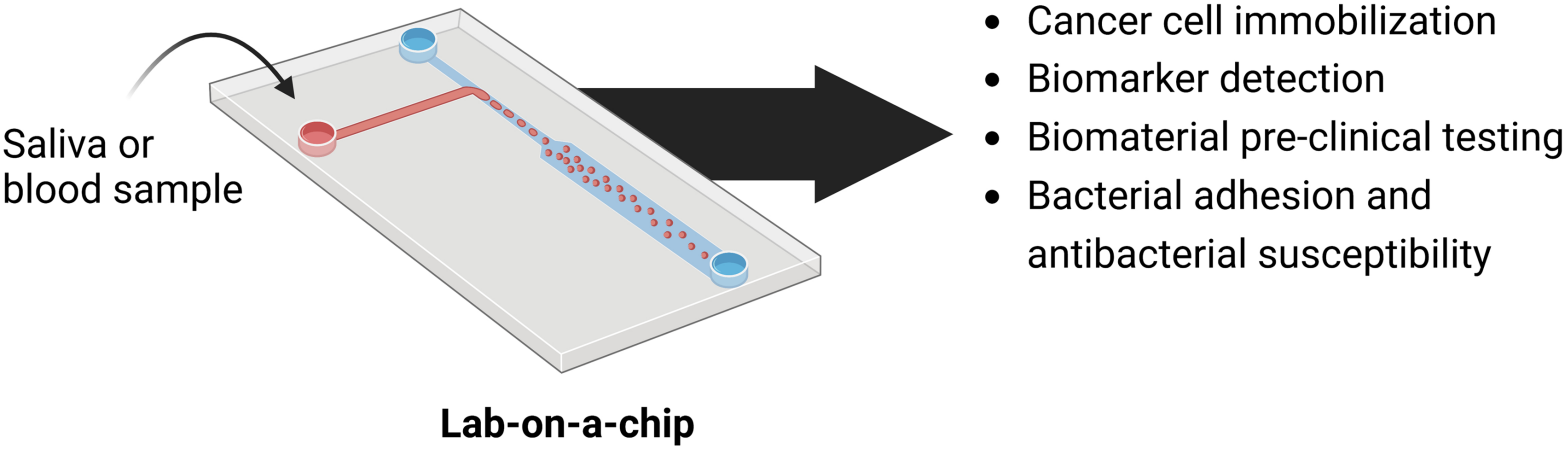
Summary of lab-on-a-chip approaches currently being explored for pre-clinical and clinical applications in dentistry. Microfluidics-based lab-on-a-chip systems are expected to serve as low-cost diagnostic platforms for saliva and blood screening against cells and biomarkers of interest. Furthermore, these microchips have shown great potential for the easy and cost-effective pre-clinical evaluation of the cytotoxic and antibacterial properties of novel dental biomaterials.

As discussed throughout this review, the use of microfabrication approaches is slowly gaining traction within dental research and clinics. Although these approaches continue to be mostly experimental at the moment, their use in clinical diagnostics and treatment is expected to increase in the future with waning costs and increasing ease of use of microfabrication methods. For example, the microfabrication of functionalized biomaterials for dental applications is an interesting avenue for the development of new, smart biomaterials with high clinical predictability. Particularly for the oral cavity, it is of interest to explore microstructures that could promote host cell adhesion and differentiation while simultaneously deterring bacterial adhesion and biofilm formation (102, 103). This would be highly relevant for the regeneration of periodontal defects in anatomical regions where biofilms play a crucial role in health and disease. The use of electrospinning for the construction of bioactive microfabricated scaffolds for restorative dentistry is also being investigated, particularly to develop antibacterial and functional biomaterial for restorative dentistry (104–106). Additionally, the use of microfluidics and organ-on-a-chip systems could serve as a cost-effective and direct point-of-care testing option for the diagnosis of a range of oral diseases, particularly for use in remote communities and areas with limited availability of high-cost diagnostic facilities (Figure 3).

**Figure 4:**
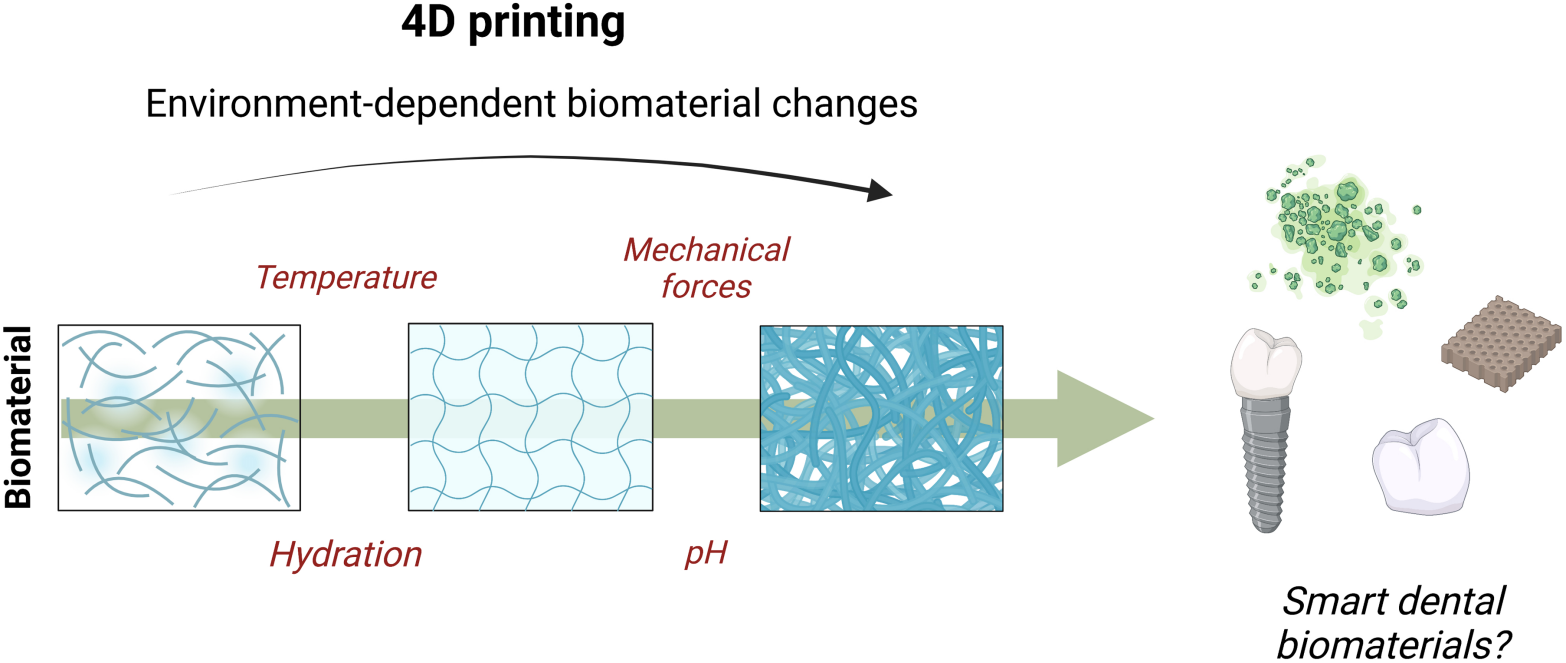
4D bioprinting as a tool for the development of cutting-edge smart dental biomaterials.

Furthermore, recent progress in the development of smart materials has potentiated the development of highly innovative approaches such as 4D bioprinting (Figure 4) (107). Unlike 3D printing, 4D printing fabricates pre-programmable biological constructs capable of actively altering their shape in response to surrounding environmental changes (108). The potential use of these smart materials for the restoration of periodontal osseous defects and caries-affected teeth could aid the rehabilitation of oral diseases in a personalized medicine approach. Moreover, the combination of advanced imaging techniques with 3D and 4D printed biomaterials could provide fast and customized surgical reconstruction for patients with craniofacial trauma, improving emergency care and long-term repair and regeneration in these individuals.

## 5. Conclusions

In summary, microfabrication is currently being used to explore many crucial physiological and pathological processes across an important number of dental disciplines, and utilized in a wide range of *in vitro* setups to study the interaction of biomaterials with host cells and microbiome components. To date, most of these approaches are not yet available in clinics but are expected to quickly gain traction due to their rapid development and promising *in vitro* and pre-clinical results. Currently, some clinical uses of 3D bioprinting are available, although the range of applications is expected to increase rapidly in the coming years to include smart-biomaterial printing, point-of-care diagnostics, and novel restorative materials for soft and hard tissue regeneration.
